# Isothermal circular strand displacement–based assay for microRNA detection in liquid biopsy

**DOI:** 10.1007/s00216-022-04228-8

**Published:** 2022-07-26

**Authors:** Noemi Bellassai, Roberta D’Agata, Giuseppe Spoto

**Affiliations:** 1 Dipartimento di Scienze Chimiche, Università degli Studi di Catania, Viale Andrea Doria 6, 95125 Catania, Italy; 2 Consorzio Interuniversitario “Istituto Nazionale Biostrutture E Biosistemi”, c/o Dipartimento di Scienze Chimiche, Università degli Studi di Catania, Viale Andrea Doria 6, Catania, Italy

**Keywords:** MicroRNA, Liquid biopsy, Isothermal amplification, Nucleic acid amplification, Microfluidic devices, Osteoarthritis

## Abstract

Extracellular miRNAs are promising targets for developing new assays for the early diagnosis and prognosis of diseases based on liquid biopsy. The detection of miRNAs in liquid biopsies is challenged by their short sequence length, low concentration, and interferences with bodily fluid components. Isothermal circular strand displacement polymerization has emerged as a convenient method for nucleic acid amplification and detection. Herein, we describe an innovative strategy for microRNA detection directly from biological fluids based on hairpin probe–assisted isothermal amplification reaction. We designed and optimized the assay to detect target analytes in 1 µL of the complex media’s biological matrix using a microfluidic device for the straightforward analysis of multiple samples. We validated the assay to detect circulating miR-127-5p in synovial fluid, recently indicated as a predictive biomarker for osteoarthritis disease. The combined use of a mutant polymerase operating with high yield and a primer incorporating locked nucleic acid nucleosides allowed detection of miR-127-5p with 34 fmol L^−1^ LOD. We quantified circulating miR-127-5p directly in synovial fluid, thus demonstrating that the assay may be employed for the convenient detection of 4.3 ± 0.5 pmol L^−1^ concentrated miRNAs in liquid biopsy samples.

**Graphical abstract:**

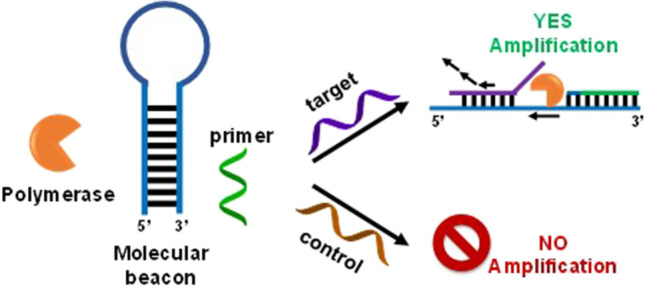

## Introduction

MicroRNAs (miRNAs) are endogenous single-stranded non-coding RNA sequences (18–25 nucleotides) interfering with the post-transcriptional regulation of gene expression [1]. They play a critical role in various cellular processes [2, 3] and tune cellular responses to environmental stresses such as DNA damage, hypoxia, and oxidative stress. miRNA aberrant expression level is associated with the pathogenesis of various diseases [4–6]. For this reason, extracellular miRNAs are promising targets for the development of new analytical tools for early diagnosis and prognosis of diseases based on liquid biopsy [7], i.e. the direct detection of biomarkers in body fluids such as serum [8], plasma [9], saliva [10] urine [11], and cerebrospinal fluid [12].

The challenging detection of miRNAs in liquid biopsies results from constraints introduced by their short sequence length, low concentration (from fM to pM in plasma) [13], and interferences caused by the complex composition of the body fluid [14, 15]. Reverse transcription-quantitative polymerase chain reaction (RT-qPCR) is the standard gold method for miRNA detection [16]. Although offering high sensitivity and specificity in miRNA detection, RT-qPCR shares with the other PCR-based methods limitations introduced by laborious and time-consuming sample processing requirements, including the isolation of the total RNA from the biological sample, thermal cycling setup, and sample contamination risk. Isothermal amplification methods such as loop-mediated amplification (LAMP) [17], exponential amplification reaction (EXPAR) [18, 19], strand displacement amplification (SDA) [20], and catalytic hairpin assembly (CHA) [21] have been investigated to overcome some of the limitations of PCR-based methods [22, 23].

**Fig. 1:**
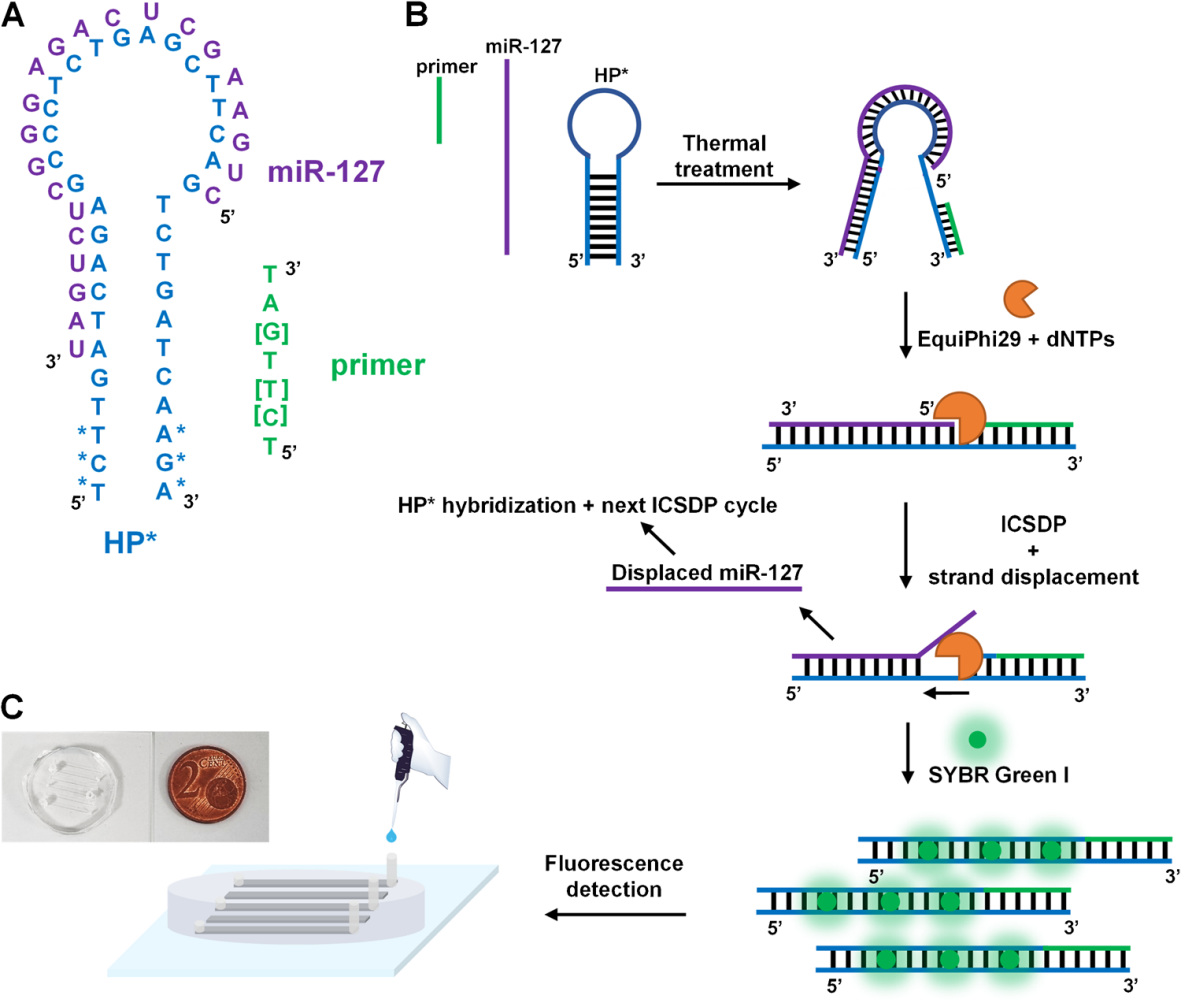
**A** Sequence of HP* hairpin probe. Sequences of miR-127 and the primer are also shown. Square brackets indicate locked nucleic acid (LNA) nucleosides. An asterisk identifies phosphorothioate (PTO) nucleotides (Table 1). **B** Schematic representation of the ICSDP-based detection of miRNA. **C** A microfluidic device is used for fluorescence detection from 1 µL of the sample volume

Isothermal circular strand displacement polymerization (ICSDP) offers a more straightforward and rapid strategy for detecting nucleic acids than other isothermal methods [24]. ICSDP uses a hairpin probe (HP* in Fig. 1A) with a target-specific stem-loop structure [25–27], a primer complementary to a portion of the 3′end sequence of HP*, and DNA polymerase with strand displacement activity (Fig. 1).

The amplification reaction occurs when the target interacts with HP*, thus triggering a conformational change of the stem-loop structure, facilitating primer annealing. Then, DNA polymerase extends the primer and displaces the hybridized target, making it available for a subsequent cycle.

Although straightforward and cost-effective, isothermal amplification methods may lose assay sensitivity and specificity. For example, non-specific amplification reactions may occur at low temperatures due to the stabilization of secondary structures or primer dimers [28]. Primers, probes, and templates bearing appropriately modified nucleotides can better control the specificity and stability of nucleic acid hybridization reaction than conventional oligonucleotides. The modified nucleotides avoid non-specific enzymatic activity, lower detection limits, contribute to optimizing the reaction temperature setting, and reduce non-specific signals [28]. In this perspective, locked nucleic acids (LNA) offer higher duplex stability and improved mismatch discrimination performance than conventional nucleic acid sequences [29–31]. Phosphorothioate nucleotides (PTO), instead, have been shown to provide resistance to nuclease activity [32] and increased efficiency in the formation of intermediate products during LAMP reaction [33] and other isothermal amplification reactions [34–36].

The confinement in microfluidic devices of the sample may represent another pivotal element for circulating miRNAs analysis, especially when a small volume of biological fluid is available [24, 37]. The combination of microfluidics with isothermal amplification technologies enables a dramatic decrease in reagents and sample volume, shorter analysis time, rapid heat and mass transfer due to the large surface-to-volume ratio, a reduced time for temperature stabilization, and a more uniform solution temperature with a consequently enhanced amplification yield.

A new ICSDP-based strategy for detecting miRNA circulating in biological fluids is presented. The assay uses a user-friendly microfluidic device to detect the biological fluid’s target miRNA in 1 µL. In particular, we targeted miR-127-5p (miR-127) as a predictive biomarker for osteoarthritis (OA) and developed procedures for its detection and quantification in synovial fluid [38–42].

Osteoarthritis (OA) is a long-term and degenerative musculoskeletal condition causing severe whole-joint damages [43, 44]. OA increases the risk of further morbidities such as diabetes [45] and cardiac disease, leading to increased mortality [46].

The specific mechanisms leading to OA are still not fully clarified [43, 44]. Medical treatments include non-pharmacological, pharmacological, and invasive surgical treatments [47, 48], which still cannot prevent the occurrence and progression of the disease. The overall cost for OA treatments has been estimated at 19,000 €/year per patient [49]. Such a cost may be linked to the failure of currently available tools for early diagnosis, providing the best opportunity to prolong the patients’ healthy years of life [50].

miRNAs are crucial in regulating several biochemical pathways implicated in OA development and progression [51], including chondrocyte apoptosis [52] and proliferation [18], cartilage extracellular matrix (ECM) metabolism, and inflammatory response [53–55]. Various miRNAs have been identified as potential OA biomarkers available in liquid biopsies such as serum [39, 56], plasma [38, 57], and synovial fluid [38–42]. Among different body fluids, synovial fluid could better clarify OA pathogenesis. Synovial fluid miRNAs help identify the earliest pathophysiological changes related to the specific degenerative musculoskeletal condition [38] and contribute to differentiating between the early- and late-stage OA disease [41, 42]. Detecting different sets of miRNA directly in the synovial fluid would facilitate early OA diagnosis and limit further damage to articular cartilage. In particular, miR-127-5p is downregulated in OA cartilage. It tunes matrix metallopeptidase 13 (MMP-13) concentration in human chondrocytes by regulating the osteopontin expression level involved in OA pathogenesis [42] and suppressing IL-1b-induced MMP-13 production [58]. So far, few reports have described miRNA detection in synovial fluids by RT-qPCR combined with cumbersome pre-analytical treatments of the biological sample [38, 42].

The detection method here described comprises a hairpin probe bearing PTO nucleotides, a mutant EquiPhi29 DNA polymerase with extremely high yields of amplified nucleic acid sequences and strand displacement capacity even at relatively low temperatures (30 °C) [59], a primer sequence including LNA nucleosides, and SYBR Green I dye to improve the assay sensitivity.

The method allowed us to quantify miR-127-5p in synovial fluid, thus demonstrating the possibility to use it for detecting miRNA biomarkers in liquid biopsy. A streamlined microfluidic device allowed us to perform the assay with 1 µL of sample volume, thereby ensuring the minimal invasiveness of the diagnostic approach.

## Material and methods

### Chemicals and materials

Experiments have been performed using ultra-pure nuclease-free water (Milli-Q Integral S3 system, Millipore). Tris-(hydroxymethyl)-aminomethane (TRIS), deoxynucleotides (dNTPs), MgCl_2_, KCl, and Tween 20 surfactant were purchased from Merck Sigma-Aldrich, Italy. RNaseOUT Recombinant ribonuclease inhibitor, EquiPhi29 DNA polymerase, and pyrophosphatase inorganic were purchased from Thermo Fisher Scientific Italy. Hairpin probe with PTO nucleotides for miR127 (HP*), target and scrambled miRNA oligonucleotides, and the unmodified primer were acquired from Eurofins Genomics, Belgium (Table 1, Fig. 1A). Eurogentec, Belgium, provided PTO and LNA modified primers (Table 1, Fig. 1A). We predicted nucleic-acid-sequence folding and hybridization using UNAFold web server. Lee Biosolutions, USA, provided synovial fluid (LOT: 01D3489 sample) from pooled human donors with inflamed joints. SYLGARD™ 184 Silicone Elastomer Kit for microfluidic device fabrication was provided by Dow Corning, USA.

**Table 1:** Sequences and acronyms of hairpin probe, primer, target, and control miRNA sequences used for ICSDP detection

Description	Acronym	Sequence (5′ →3′)	*T*_m_ (°C)^a^
Hairpin probe with PTO nucleotides for miR-127^b^	HP*	T*C*T*TGATCAGAGCCCTCTGAGCTTCAGTCTGATCAA*G*A*	71.6
Primers w/o PTO and/or LNA modifications^c^	P* [P], [P*]	TCTTG*AT T[C][T] T [G]AT, T[C][T] T [G]*AT	18.0 23.0
Target hsa-miR-127-5p	miR-127	CUGAAGCUCAGAGGGCUCUGAU	62.1
Control miRNA	miR-CTR	AGCUACAUUGUCUGCUGGGUUUC	60.6

^a^*T*_m_ for LNA primers was estimated by LNA Oligo Design Tools and Calculators of GeneGlobe (https://geneglobe.qiagen.com/mx/explore/tools/tm-prediction/form).

^b^Underlined sequences show the hairpin stems. Bold letters highlight the sequence complementary to the primer.

^c^ “[]” = locked nucleic acid (LNA) nucleoside; “*” = phosphorothioate (PTO) nucleotide.

### Fabrication of microfluidic devices

Microfluidic devices in polydimethylsiloxane (PDMS) were fabricated by replica moulding as described elsewhere [60]. PDMS channels were created through replication from masters in polyvinyl chloride (80 μm depth, 400 μm width, 1.4 mm length), as already reported [60]. We obtained replica moulding by mixing PDMS curing agent and prepolymer at a 1:10 ratio. The viscous mixture was degassed under vacuum and poured onto the master for polymerization (at least 48 h) at room temperature. The mould was peeled off the master surface, washed carefully with ultra-pure water and ethanol, and dried before use. We inserted Masterflex Transfer Tubing (in polytetrafluoroethylene (PTFE). ID 0.794 mm; OD 1.588 mm. Cole-Parmer, USA) in holes drilled into the PDMS device and used as the inlets. Oxygen-plasma etching was performed to obtain the irreversible adhesion of PDMS moulds with microscope cover glasses (Femto Diener Electronics plasma cleaner, 40 kHz generator, 30 s, 30 W Diener Electronic GmbH + Co. KG., Germany). Before using it, the microfluidic device was heated at 80 °C for 20 min and left it at room temperature for at least 24 h to strengthen the PDMS/glass binding.

### Hairpin probe–assisted ICSDP amplification

0.1 nmol L^−1^ HP*, 0.1 µmol L^−1^ primer (P*, [P], or [P*]), and an indicated amount of miRNA sequences (miR-127 or miR-CTR, concentration from 0.0 to 0.50 pmol L^−1^) in the ICSDP amplification buffer (10.0 mmol L^−1^ MgCl_2_, 66.0 mmol L^−1^ KCl, 0.1% Tween 20, and 33.0 mmol L^−1^ Tris − HCl, pH 8.0) were incubated at 95 °C for 5 min before the isothermal amplification. Then, the mixture was cooled at 45 °C for 10 min and kept it at 4 °C for 10 min to promote the primer’s annealing (Fig. 1B). We performed the ICSDP amplification (2 h at 30 °C) by adding 100 μmol L^−1^ deoxynucleotide triphosphates (dNTPs) mix, 0.4 U μL^−1^ EquiPhi29 DNA polymerase, 0.01 U μL^−1^ inorganic pyrophosphatase, and 40 U RNAse OUT inhibitor in 20 μL as the final volume of the reaction mixture (Fig. 1B). Primers with or without PTO and/or LNA modifications were used to optimize the ICSDP amplification process (Table 1). We also investigated the role of the concentration ratio between the primer and HP* (from 0:1 to 10000:1).

### Fluorescence detection of miRNAs in buffer

After ICSDP amplification, we stained amplicons with 1 μL of 100 × SYBR Green I for 15 min at room temperature (Fig. 1B) and introduced 1 μL of the stained mix into the microfluidic device for the detection of the emitted fluorescence (Fig. 1C). A Leica DM IL LED inverted microscope equipped with a Leica DFC monochrome 7000 GT camera and a Leica EL6000 external light source (mercury metal halide bulb) with a 497 nm excitation wavelength and filter selected emission wavelength of 520 nm (Leica Microsystems, Germany) was used for fluorescence imaging. We probed the fluorescence emitted through the microfluidic device’s glass substrate, whereas the fluorophore excitation was performed through PDMS. Fiji ImageJ 1.52 software allowed the analysis of the recorded images and the quantification of fluorescence signals. We selected a region of interest (ROI) from the acquired image and calculated the average intensity of the fluorescence signal detected from ROI. We referenced fluorescence intensity values as (*F* − *F*_0_)/*F*_0_, where *F*_0_ and *F* are the fluorescence intensities in the absence and presence of the relevant miR sequence, respectively.

### Detection of miR-127 in human synovial liquid

Synovial liquid from human donors with inflamed joints was centrifuged (4 °C, 13800 rpm, 30 min) and stored at − 20 °C for 24 h. Then, we thawed it and centrifuged it again for 10 min (4 °C, 13800 rpm).

Before performing the isothermal amplification, 0.1 nmol L^−1^ HP*, 0.1 µmol L^−1^ [P] primer, an indicated amount of spiked miR-127 (concentration ranging from 0.0 to 0.30 pmol L^−1^), 40 U RNAse OUT inhibitor, and 2 μL of the synovial liquid sample, indicated as the constant sample aliquot in both un-spiked and spiked solutions, in ICSDP buffer were incubated at 72 °C for 10 min. Then, the mixture was cooled at 45 °C for 10 min and kept it at 4 °C for 10 min to promote the primer’s annealing. The ICSDP amplification was performed for 2 h at 30 °C by adding 100 μmol L^−1^ deoxynucleotide triphosphates (dNTPs) mix, 0.4 U μL^−1^ EquiPhi29 DNA polymerase, and 0.01 U μL^−1^ inorganic pyrophosphatase in 20 μL as the final volume of the reaction mixture. The dilution ratio of the synovial fluid in the ICSDP reaction mixture was 1:10, and the volume ratio of reagents in the ICSDP mixture was optimized according to the laboratory settings.

After ICSDP amplification, we added 1 μL of 5 × SYBR Green I to 20 μL as reaction volume and incubated the mixture for 15 min at room temperature to stain amplificons. Lately, we injected 1 μL of the stained ICSDP mixture into the microfluidic device for fluorescence detection. We determined the concentration of circulating miR-127 in synovial fluid by the standard addition method.

## Results and discussion

### Hairpin probe design

A proper design of the hairpin probe is mandatory to achieve an efficient ICSDP amplification triggered by the hybridization of the miRNA target. We designed HP* to comprise a sequence complementary to miR-127. Such a sequence corresponds to the loop and a portion of the 5′-end strand of the stem of HP* (Fig. 1A). The 3′-end strand of the stem is instead complementary to the primer (P*, [P], or [P*]). HP* stem consisted of an 11 nt-long sequence, whereas a 7 nt-long primer was used for ICSDP amplification (Table 1, Fig. 1A). HP* stem sequence should stabilize the hairpin configuration in the presence of the primer alone, thus preventing unspecific amplification reactions triggered by HP*/primer interactions. For this reason, we designed a hairpin probe sequence with *T*_m_ higher than 45 °C to keep it stable under the ICSDP thermal conditions in the absence of the target sequence.

The ICSDP amplification here described exploits the enzymatic activity of EquiPhi29 DNA polymerase. We selected such polymerase for its high processivity and vigorous strand displacement activity. Both features translate into a target amplification with high yields even at relatively low temperatures (30 °C) [59]. We introduced three PTO-modified nucleotides at both 5’ and 3’ termini of the sequence to protect HP* from the 3′ →5’ exonuclease activity of EquiPhi29 DNA polymerase. As already pointed out, we selected miR-127 as the target to be detected in the synovial fluid for early OA diagnosis based on liquid biopsy analysis.

### Optimization of hairpin probe assisted-ICSDP reaction

The first step of the assay is the thermal unfolding of HP* obtained heating at 95 °C (5 min) a mixture comprising HP*, the primer, and miR-127 or miR-CTR in ICSDP buffer. The unfolded HP* structure favoured the hybridization of miR-127 when the temperature was decreased to 45 °C (10 min). The annealing of the primer with the unfolded HP* structure was promoted during the final thermal step (4 °C for 10 min) (Fig. 1B). After the thermal treatment, the ICSDP reaction was activated by adding EquiPhi29 DNA polymerase, dNTPs nucleotides, RNAseOUT inhibitor, and inorganic pyrophosphatase to the solution and incubating the mixture at 30 °C for 2 h. The RNAseOUT inhibitor and pyrophosphatase prevented the degradation of miRNA sequences and pyrophosphate accumulation during the amplification, respectively.

The final products of the ICSDP reaction were quantified by detecting the fluorescence produced after incubation with SYBR Green I dye (15 min, room temperature, Fig. 1B). In particular, a manageable and straightforward microfluidic device was exploited to detect fluorescence signals from only 1 µL of the ICSDP mixture, defined as the injection volume needed for each analysis (Fig. 1C).

**Fig. 2:**
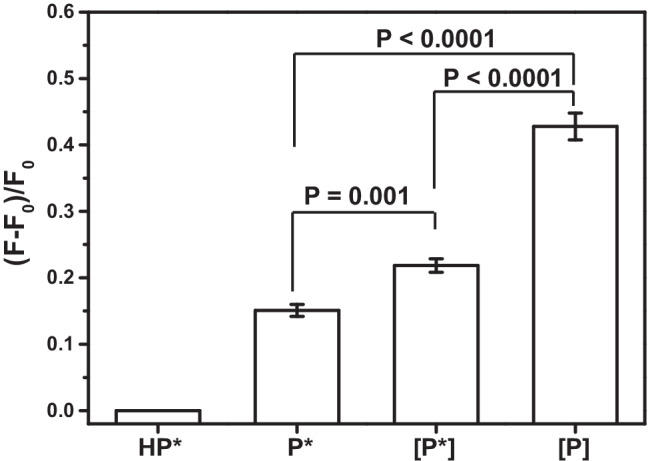
Isothermal amplification reaction of miR-127 (0.5 pmol L^−1^) assisted by HP*. Fluorescence data were initially collected by conducting the assay with no primer (HP*), and then by using primers with differences in their structures, i.e. with a PTO nucleotide (P*), with a PTO nucleotide and LNA nucleosides ([P*]), and with only LNA nucleosides ([P]) (Table 1), respectively, to identify the best performing primer. The experiments were conducted with a 1:1 concentration ratio between primer and HP* (0.1 µmol L^−1^). (*F* − *F*_0_)/*F*_0_ values were evaluated after miR-127 detection. Error bars show the standard deviation of values referring to three independent ROIs. The two-tailed *t*-test, level 95%, *p*-value = 0.001 for P*/[P*] results and two-tailed *t*-test, level 95%, *p*-value < 0.0001 for P*/[P] and [P*]/[P] results confirm that the data are statistically different

We tested three types of primers, i.e. with a PTO nucleotide (P*), a PTO nucleotide and LNA nucleosides ([P*]), and with only LNA nucleosides ([P]) (Table 1), respectively, to identify the best performing primer structure. With this aim, we initially recorded the fluorescence signal produced after conducting the ICSDP reaction with no primer, and, then, we tested each primer against the ICSDP amplification of miR-127 (0.5 pmol L^−1^) by performing experiments with a 1:1 concentration ratio between primer and HP* (0.1 µmol L^−1^) (Fig. 2).

As shown in Fig. 2, [P] provided the best performance and produced the most intense signals after the ICSDP amplification of miR-127. These experiments demonstrate that the three LNA nucleosides included in [P] favour the annealing of the primer with the HP*-miR-127 dimer even at low temperature (30 °C) despite the small size of the primer.

### Analytical performances of ICSDP assay

#### Amplification efficiency, detection limit, and target discrimination

**Fig. 3:**
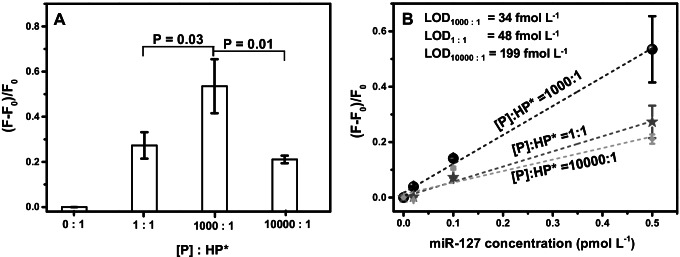
**A** ICSDP assays conducted with 0.5 pmol L^−1^ miR-127 concentration and [P] primer (with LNA nucleosides). Different [P]:HP* concentration ratios are studied (0:1—i.e. no primer, 1:1, 1000:1, and 10000:1. 1 corresponding to 0.1 nmol L^−1^). Error bars show the standard deviation of values referring to three independent ROIs. Two-tailed *t*-tests, level 95%, confirm that data are statistically different. **B** ICSDP assays conducted with different miR-127 concentrations (0, 0.02, 0.1, and 0.5 pmol L^−1^) and different [P]:HP* concentration ratios. 1000:1 [P]:HP* concentration ratios provides the most intense signals and the best linear correlation (*r*^2^ = 0.998, to be compared with *r*^2^ = 0.992 for 1:1 ratio and *r*^2^ = 0.880 for 10000:1 ratio)

The primer:HP* concentration ratio also affects the hybridization efficiency. For this reason, we considered fluorescence signals detected after the ICSDP amplification of miR-127 (0.5 pmol L^−1^) when using three [P]:HP* concentration ratios (i.e. 1:1, 1000:1, and 10000:1. 1 corresponding to 0.1 nmol L^−1^) and compared them with the signal produced by HP* in the absence of the primer (0:1). As shown in Fig. 3A, the 1000:1 concentration ratio produced the most intense signals. Interestingly, the highest [P]:HP* ratio (10000:1) investigated disfavours the amplification compared to 1:1 and 1000:1 ratios (Fig. 3A). Such peculiar evidence may result from the mild self-dimerization of primers favoured at the highest concentration. Such a dimeric structure includes four matching base pairs (T-A, A-T, C-G, G-C), resulting in a low Δ*G* calculated value (− 1.57 kcal/mole) [61]. The formation of such homo-dimer reduces the availability of primer sequences for hairpin probe hybridization, thus affecting the amplification efficiency.

For a more comprehensive evaluation of the incidence of [P]:HP* concentration ratio on the assay sensitivity, we compared data obtained from experiments performed with different miR-127 concentrations (0, 0.02, 0.1, and 0.5 pmol L^−1^). Figure 3B shows the linear correlation between the intensity of the detected signals and miR-127 concentrations detected for the different [P]:HP* ratios. The 1000:1 ratio provided the best responses throughout the investigated concentration interval by providing LOD = 34 fmol L^−1^ (3σ method), whereas 48 fmol L^−1^ and 199 fmol L^−1^ LODs were obtained for 1:1 and 10000:1 ratios, respectively. Notably, the described approach improves the detection limit by one order of magnitude compared with the assay coupling multiple isothermal amplifications elsewhere described [62], and by two orders of magnitude compared with digital flow cytometry-ligation rolling circle amplification [63]. LOD we obtained for detecting miR-127 is similar to those obtained with rolling circle amplification–derived [64, 65] and LAMP[17] methods while offering more straightforward procedures for the amplification and detection. The method’s performances are related to HP* peculiar design and inclusion of PTO nucleotides (Table 1), high efficiency of the EquiPhi29 DNA polymerase-based reaction, and LNA nucleotides’ presence in the primer sequence.

When combined with a microfluidic platform for the signal readout, the hairpin probe–assisted ICSDP amplification performs a rapid, simple, and effective microRNA detection with analytical features relevant for osteoarthritis diagnosis. Microfluidic chip offers several assets over conventionally sized systems, including less volume of the clinical sample, low reagent consumptions, improved temperature control, and multiplexed target detection. Conventional sample cells using larger sample volumes take advantage of the more significant light path for fluorescence measurement that could enhance the analytical sensitivity but exclude all the benefits related to exploiting the microfluidic device here proposed. As already demonstrated [66, 67], specific microfluidic platforms could integrate the hairpin probe assisted-ICSDP amplification with the target detection for lab-on-a-chip applications.

**Fig. 4:**
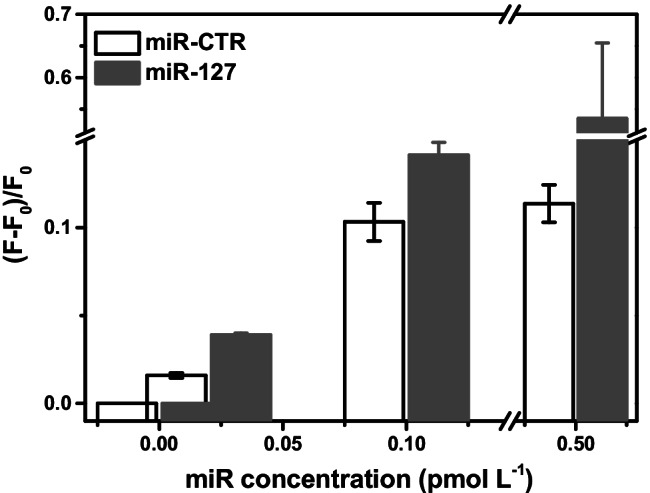
ICSDP assays conducted with miR-127 or miR-CTR at 0, 0.02, 0.1, 0.5 pmol L^−1^ concentration. The experiments were conducted using [P] primer with 1000:1 [P]:HP* concentration ratio (1 corresponding to 0.1 nmol L^−1^). Error bars show the standard deviation of values referring to three independent ROIs

We investigated the capacity of the assay to discriminate between miR-127 and miR-CTR within a clinically relevant concentration range (0, 0.02, 0.1, 0.5 pmol L^−1^) (Fig. 4) to investigate the assay’s performances further.

As shown in Fig. 4, we achieved discrimination of signals referring to target (miR-127) and control (miR-CTR) sequences throughout the investigated concentration range.

#### Quantification of miR-127 in synovial fluid

The results from the already presented experiments prompted us to apply the ICSDP assay to detect miR-127 in the synovial fluid obtained from pooled human donors with inflamed joints to demonstrate the optimized assay’s applicability for OA diagnosis disease through liquid biopsy.

We performed the experiments by analyzing un-spiked and miR-127 spiked synovial fluid samples, with no preliminary isolation of total RNA from the samples. Spiked samples were obtained by adding increasing volumes of a standard miR-127 solution, and all samples (including un-spiked samples) were diluted to reach the same final volume to comply with requirements for the standard addition method. We performed the assay by adding HP*, [P], RNAse OUT inhibitor, and miR-127 (final concentration ranging from 0.0 to 0.3 pmol L^−1^) to tenfold diluted synovial fluid previously thawed and centrifuged (see Material and methods section). The mixture was thermally treated as already described to promote the HP* unfolding, HP*/miR-127 hybridization, and [P] annealing. The ICSDP amplification was performed for 2 h at 30 °C by adding dNTPs mix, EquiPhi29 DNA polymerase, and inorganic pyrophosphatase into the final reaction mixture, and fluorescence was detected using 1 μL of the solution obtained after staining the amplicons with SYBR Green I.

**Fig. 5:**
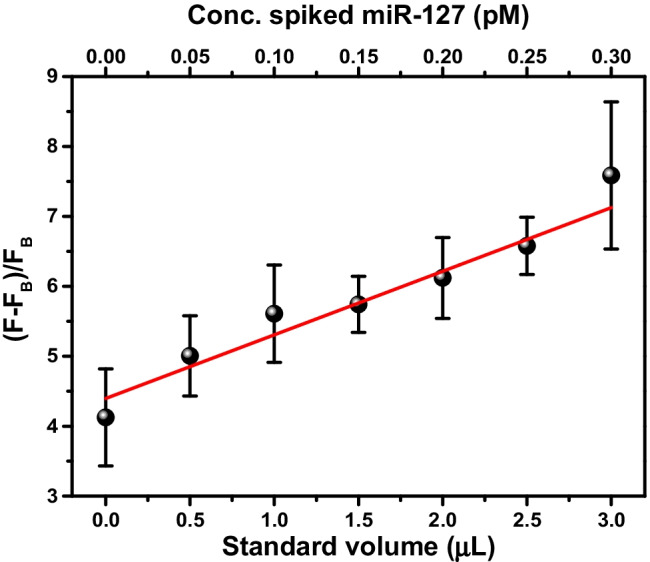
Standard addition method for circulating miR-127 detection directly in OA patients. The fluorescence intensity detected from synovial fluid samples (*F*) is referenced to the background signal (*F*_B_) detected outside the microfluidic channel. (*F* – *F*_B_)/*F*_B_ refers to fluorescence detected from miR-127 spiked 10% diluted synovial fluid samples after ICSDP amplification. Error bars indicate the standard deviation of three independent ROIs in the microfluidic channel. The linear regression equation after standard additions provided the equation *y* = 4.3(2)*x* + 1.0(1) (*r*^2^ = 0.95). The standard addition method provided a miRNA concentration in the analyzed synovial fluid of 4.3 ± 0.5 pmol L.^−1^ (standard deviation)

Figure 5 shows the linear correlation (*r*^2^ = 0.95) between the fluorescence response and the concentration of miR-127 spiked in the synovial fluid, confirming the assay’s capacity to perform correctly also when operating in the synovial fluid complex matrix. The un-spiked synovial fluid produces relatively high fluorescence signals due to endogenous miR-127 and the unspecific signals generated by the complex matrix. For this reason, we quantified the endogenous miR-127 in the synovia sample using the standard addition method that provided 4.3 ± 0.5 pmol L^−1^ concentration. Such a concentration is in line with the reported downregulated miRNA levels in OA patients [38].

These results confirm that the proposed ICSDP approach assisted by HP* and modified LNA primer sequences can directly quantify miRNAs in synovia with good sensitivity and a straightforward approach compared to current standard methods, thus holding potential for further applications in clinical diagnosis of miRNA-related pathological conditions.

## Conclusion

In summary, we have developed a hairpin probe–based isothermal strand displacement polymerization method to detect miRNAs and applied it to the quantitive detection of osteoarthritis-related miR-127 in synovial fluid. We optimized the assay conditions by identifying (i) optimal HP* and primer structures comprising PTO nucleotides and LNA nucleotides and (ii) primer:HP* concentration ratio, and (iii) efficient and convenient polymerase. The optimized conditions enable carrying out the ICSDP-based assay at a low temperature (30 °C) and in a relatively short time (2 h). We designed and optimized the assay to skip procedures for RNA isolation from the patient’s synovial fluid and simplify pre-analytical procedures. We detected the fluorescence signals using 1 µL of sample and a simple, cost-effective and reusable microfluidic device.

The assay allows for sensitive detection of miR-127 with a detection limit of 34 fmol L^−1^ in ICSDP buffer. The optimized assay can detect circulating miR-127 directly in synovial fluid at 4.3 ± 0.5 pmol L^−1^ concentration, with no need for preliminary isolation of total RNA from the body fluid and leading to significant advantages for the straightforward detection and quantification of nucleic acid biomarkers circulating in bodily fluids with applications in early clinical diagnosis and personalized medicine based on liquid biopsy.

## Data Availability

All the data are described within the manuscript. The raw data and metadata analyzed during the current study are available from the corresponding author on request.
